# Recombinant Antibody Vaccines for Targeted Antigen Delivery: Design Principles and Immunological Applications

**DOI:** 10.3390/vaccines14090788

**Published:** 2026-09-09

**Authors:** Kaitlyn M. Pirogovsky, Daniel Hawiger

**Affiliations:** Department of Molecular Microbiology and Immunology, School of Medicine, Saint Louis University, St. Louis, MO 63104, USA; kaitlyn.danforth@slu.edu

**Keywords:** immunizations, monoclonal antibodies, antigen targeting, antigen delivery, dendritic cells

## Abstract

Vaccines that elicit durable immune responses require efficient delivery of antigen to antigen-presenting cells (APCs), in combination with appropriate contextual signals that promote immunity. Recombinant antibody vaccines are a flexible platform for antigen delivery in which monoclonal antibodies (mAbs) or antibody-derived fragments carry antigenic cargo to chosen receptors on target cells. In this review, we examine the design principles, mechanisms, and applications of recombinant antibody vaccines, with a focus on strategies targeting dendritic cells (DCs). DCs can take up, process, and present antigen, leading to effective induction of adaptive immune responses. Targeting of DCs with recombinant antibody vaccines improves the efficiency of antigen delivery and allows finetuning of responses through choice of a targeting receptor, antigen, and adjuvant. Recombinant antibody vaccines have been evaluated across a variety of immunological settings, including for vaccination against viral, bacterial, and protozoan pathogens, therapeutic cancer vaccination, and inverse vaccination to promote antigen-specific tolerance in models of autoimmune disease. Collectively, these studies demonstrate the versatile applications of recombinant antibody vaccines, although continued work is needed to define the elements most likely to support translation to the clinic. Overall, recombinant antibody vaccines provide a modular platform for targeted antigen delivery and represent a promising strategy for design of next-generation vaccines.

## 1. Vaccine Technologies and the Importance of Dendritic Cells in Vaccination

Vaccines confer lasting immunological protection against an infection or disease upon subsequent exposure to an antigen [1]. While vaccines most commonly are designed to induce immunity against infectious pathogens, vaccines can also be developed to promote antitumor immunity or to prevent autoimmune disease [2,3,4,5,6]. There are many different vaccine types, each with their own set of benefits and drawbacks that must be considered during the vaccine design process. A classical vaccine type is a live attenuated vaccine, in which a pathogen is used directly, but in a form in which virulence is significantly reduced [7]. Many licensed vaccines use this approach, and the intact ability of the pathogen to replicate contributes to vaccine efficacy; however, these vaccines do carry the risk of causing disease, especially in immunocompromised individuals [1,7]. In contrast, inactivated or killed vaccines cannot cause the diseases they target because they cannot replicate, but these vaccines have a limited ability to initiate cellular immunity and may require booster doses due to decreased immunogenicity [7,8]. Outer membrane vesicle (OMV) vaccines similarly employ non-replicating pathogen-derived material but consist of nano-sized vesicles shed by Gram-negative bacteria [9]. OMV vaccines can present multiple antigens at once and are intrinsically immunogenic but are limited by manufacturing variability and lipopolysaccharide-associated toxicity [9]. Other approaches leverage only certain antigens, such as protein or other subunit vaccines. These are less reactogenic than live-attenuated or inactivated vaccines, but require adjuvants to enhance the immune response and provide immunity only to the target antigens, which must be carefully selected [7]. Viral vector and nucleic acid vaccines deliver genomic material encoding target antigens, making them versatile and adaptable, as evidenced by the rapid development of mRNA-lipid nanoparticle (LNP) vaccines against severe acute respiratory syndrome coronavirus 2 (SARS-CoV-2) [1]. These approaches are relatively new, and ways to improve their efficiency, specificity, and immunogenicity are still being uncovered [1]. An approach that combines many of the benefits of these various vaccine platforms is recombinant monoclonal antibody (mAb) vaccines. mAb vaccines provide highly specific delivery of antigen to specific cellular populations, can induce cellular and humoral immunity, have demonstrated favorable safety profiles in early clinical testing, and are modular, allowing adaptation for new diseases and antigens with relative ease [10,11,12,13].

Dendritic cells (DCs) act as an essential bridge between the innate and adaptive immune systems by providing the necessary signals for T cell activation, making them a key target in many vaccination strategies [14,15,16]. Recombinant mAbs specific to DC surface receptors can act as vaccines that ferry antigen directly to DCs, enabling efficient targeting of this critical population [10,17,18,19,20,21]. DCs include two primary subtypes: plasmacytoid DCs (pDCs) and conventional DCs (cDCs). pDCs, which act as a major source of type I interferons and promote antiviral immunity, express surface markers such as B220, DC-SIGN, Ly6C, BST2, Siglec-H, and CCR9 [22,23,24]. cDCs are further split into the subsets cDC1s and cDC2s. cDC1s express the surface markers Xcr1, with certain subsets also expressing CD8α, CD24, Clec9a, DEC-205, and BTLA [25,26,27]. On the other hand, cDC2s are marked generally by expression of CD11b and CD172a (Sirpα), with some subsets also expressing CD4 and ESAM [25,26,27]. The differential expression of these surface molecules allows for mAb vaccines to target defined DC subsets, leading to specific immunological responses as outlined extensively below. In this review, we will discuss the mechanisms underlying recombinant mAb vaccines and explore current strategies for targeting antigen delivery to these distinct DC subsets, as well as the resulting immunologic outcomes.

## 2. Mechanisms of DC-Targeted Recombinant Monoclonal Antibody Vaccines

**Figure 1:**
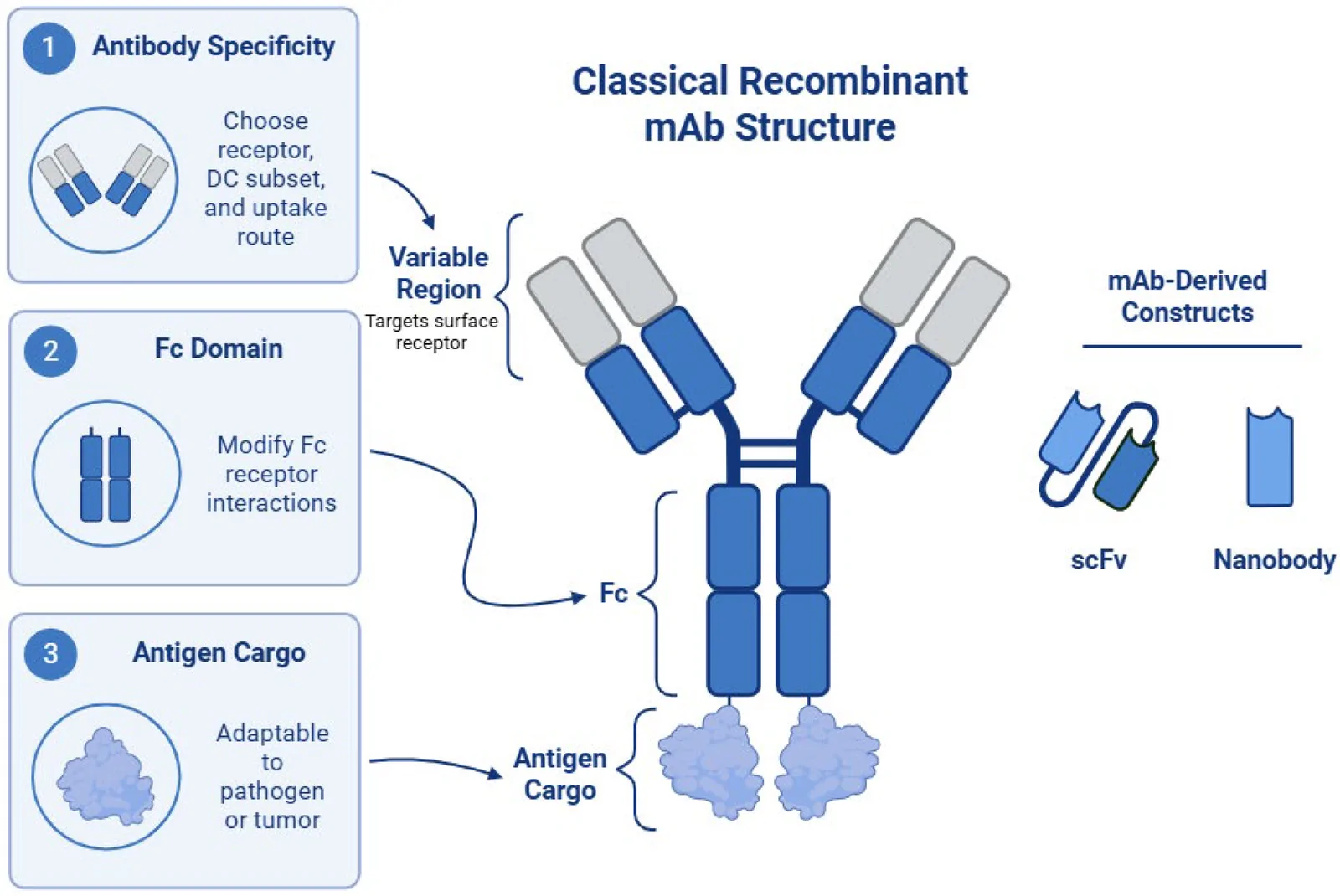
Design of recombinant monoclonal antibody vaccines.

Classically, recombinant mAb vaccines are designed as IgG-based fusion proteins, wherein the variable regions of the mAb confer specificity for a target surface receptor and the antigen is fused to the C-terminus of the constant region [17,19]. Such design allows for independent engineering of multiple features, including the antibody specificity, Fc domain, and antigen cargo. Antibody specificity can be selected to target receptors associated with particular DC subsets, uptake pathways, and immunological outcomes, and the Fc region can be modified to alter interactions with Fc receptors [28,29,30]. Similarly, the fused antigen cargo can be adapted to the pathogen or tumor of interest, allowing for adaptation across distinct immunological settings [10] (Figure 1).

Although a full-length IgG fusion protein is the classical choice for the design of recombinant mAb vaccines, there are several design choices that can be made to improve such vaccines within their specific contexts. Advantages of a full-length IgG construct include established production methods, multivalent engagement of target receptors, and an extended half-life in serum due to interactions with the neonatal Fc receptor for IgG (FcRn) [31,32,33,34,35]. However, Fc-mediated interactions, unless mitigated by mutations that disrupt binding to Fc receptors, can impact biodistribution and intracellular trafficking or lead to non-specific uptake [18,20,36,37]. Therefore, some recent strategies have leveraged antibody-derived molecules including single-chain variable fragments (scFv) or heavy-chain-only nanobodies, which can alter pharmacokinetics or further prevent undesirable Fc receptor binding [10,18,20,38,39,40,41,42,43,44,45,46,47]. Antigen linkage is another important aspect of mAb vaccine design. While many designs genetically link antigen to the C-terminus of the heavy chain, alternative approaches include genetic N-terminal fusion, chemical conjugation, site-specific enzymatic linkage, or proximity-induced conjugation [12,17,19,48,49,50]. These approaches vary in antigen stoichiometry, specificity of antigen attachment, and ease of antigen exchange, so the ideal linkage strategy depends on the intended application and desired immunological outcome.

DC-targeted recombinant mAb vaccines deliver antigen via receptor-mediated uptake pathways [10,17,51]. Upon antibody (Ab) binding to the target receptor, the Ab-antigen conjugate is internalized via clathrin-mediated endocytosis and delivered to intracellular vesicular compartments [52]. Targeting of different DC subsets and receptors results in distinct patterns of intracellular trafficking and antigen processing following internalization, ultimately impacting the induced immune response [52,53,54]. The acquired antigen is ultimately broken down by lysosomal proteases, generating peptide fragments that can be presented on MHC [55]. Such fragments may follow the conventional path for presentation on MHC class II within the endosomal compartments or, alternatively, may be presented on MHC class I in a process called cross-presentation [56,57].

Upon presentation of antigen on MHC, the resulting T cell response is shaped by T cell receptor stimulation, cytokines, and co-stimulatory signals [55,58,59]. In the steady state, DCs demonstrate tolerogenic functions, inducing T cell anergy or regulatory T cell (T_reg_) formation [10,19,60,61,62,63,64]. Additionally, DCs in the steady state can induce pre-effectors that do not initially express effector transcription factors but will more readily differentiate into effectors upon re-stimulation [63,65]. In contrast, under pro-inflammatory conditions such as viral infection, DCs become activated via pattern recognition receptor (PRR) stimulation and induce potent effector T cell responses [66,67,68,69,70]. When developing a vaccine against pathogens or cancer, adjuvants are used to engage PRRs, shifting the response from tolerogenic to immunogenic and permitting effector T cell differentiation [67,71]. In experimental recombinant mAb vaccine studies, Toll-like receptor (TLR) ligands such as polyinosinic: polycytidylic acid (poly (I:C)) and poly-ICLC or agonistic anti-CD40 antibodies are commonly used adjuvants [10,11,19,72,73,74,75,76,77,78,79]. Adjuvant selection can enhance and modulate immune activation to achieve specific outcomes [20,67,71,72,73,78].

Because DCs direct the downstream T cell response in this way, they represent attractive targets for vaccination strategies designed to elicit specific immunological outcomes. Furthermore, beyond regulating the magnitude of immune responses and promoting either tolerogenic or immunogenic outcomes, DCs influence early T cell fate decisions and differentiation states following antigen encounter [65]. A population receiving increasing attention in vaccine and immunotherapy development is stem-like T cells (T_STEM_), a long-lived, self-renewing population capable of sustaining effector T cell production [80,81,82,83,84]. While the precise mechanisms by which DCs promote T_STEM_ generation remain incompletely understood, modulating these fate decisions during T cell priming represents an important consideration in vaccine design [85].

Given the central role DCs play in initiating adaptive immune responses, numerous strategies have been developed targeting antigen delivery to specific DC subsets via recombinant mAbs to achieve distinct immunological outcomes. The first such strategy targeted the endocytic receptor DEC-205, which is expressed at high levels on cDC1s [19,86,87]. Antigens delivered via DEC-205 targeting are efficiently presented on both MHC class I and class II [19,88,89]. Initial work established that, in the steady state, antigen delivery via DEC-205 induced tolerance in CD4^+^ T cells, and later work showed that the same was true for CD8^+^ T cells [19,89]. However, when DEC-205-targeted antigen is delivered in conjunction with activating signals such as anti-CD40, the outcome is generation of effector T cells rather than peripheral tolerance [19].

In addition to DEC-205, there are other DC surface receptors that have been targeted for delivery to DCs and specific DC subsets, as extensively outlined in [10]. Another major target on cDC1s is Clec9a (also known as DNGR-1), an endocytic receptor involved in the uptake of antigen from dead and damaged cells [21,90,91]. Antigens delivered via anti-Clec9a recombinant mAbs can be cross-presented and induce potent adaptive immune responses [13,90,92,93,94,95,96]. The receptor Trem-like 4 (Treml4) has also been used for antigen targeting to cDC1s [97,98]. Langerin (CD207), which is expressed by Langerhans cells (LC) and certain cDC1s, has also been shown to successfully induce both CD8^+^ and CD4^+^ T cell responses [25,98,99,100,101]. In contrast, specific targeting to cDC2s has been accomplished via anti-dendritic cell inhibitory receptor 2 (DCIR2, also known as Clec4a4) recombinant mAbs [54,98,102,103,104,105]. Unlike cDC1-targeted approaches, targeting of DCIR2 does not strongly induce CD8^+^ T cell responses but can induce robust CD4^+^ T cell and humoral responses [36,105].

pDCs are also able to present antigens, stimulate cellular and humoral immunity, and promote immune tolerance [24]. Antigen delivery targeted to sialic acid binding Ig-like lectin H (Siglec-H) or blood dendritic cell antigen 2 (BDCA2) have been used to attenuate effector T cell differentiation and prevent the onset of autoimmunity [106,107]. BST2-targeted antigens have also been shown to be efficiently presented on pDCs and can induce protective immune responses when delivered in conjunction with adjuvant [108,109].

**Figure 2:**
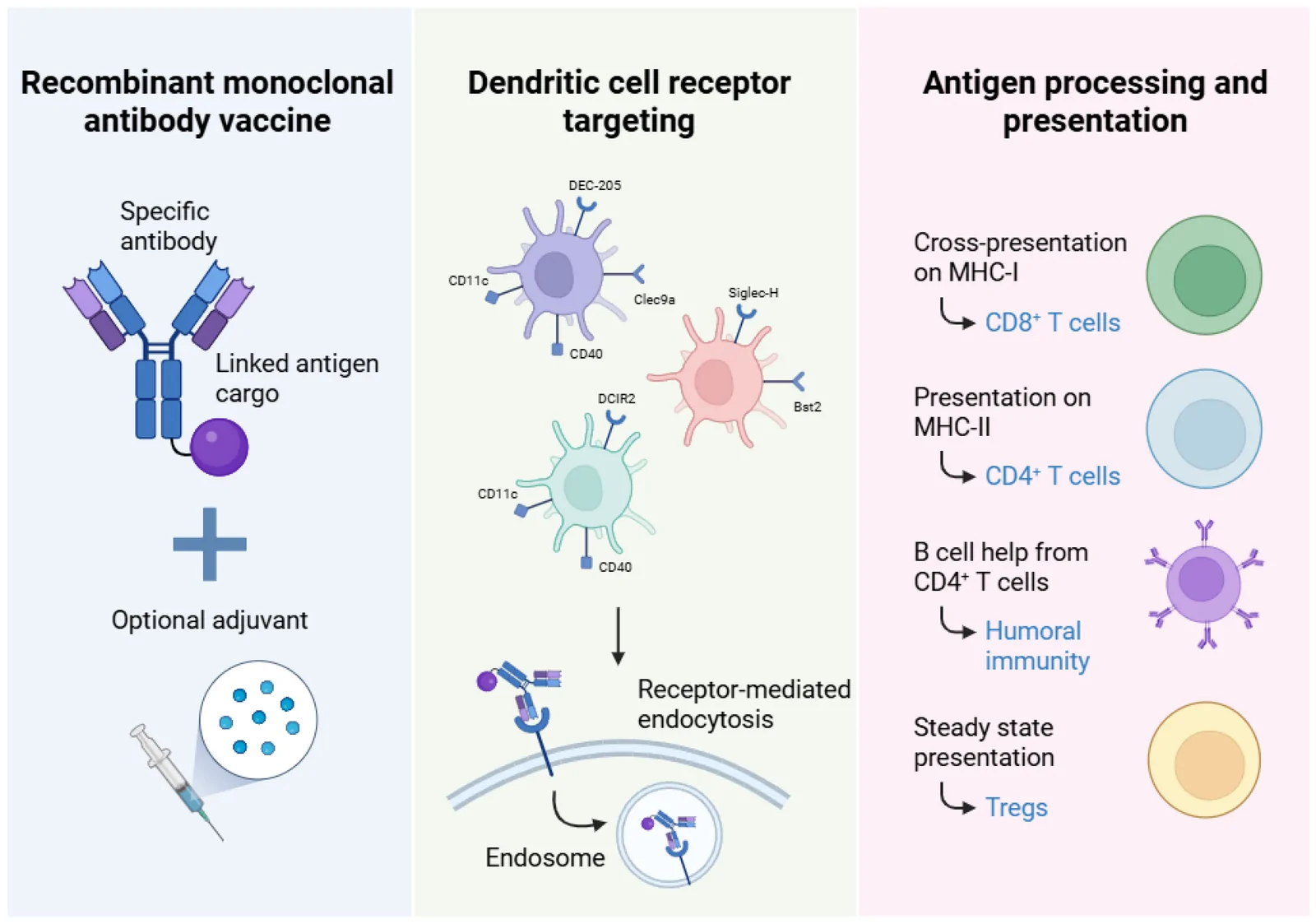
Recombinant monoclonal antibody delivery of antigen to dendritic cells (DCs).

Broad targeting of multiple DC subsets can be achieved via anti-CD11c and anti-CD40 recombinant mAbs [74,75,76,77,110,111,112,113,114]. Other strategies target molecules that may not be specifically limited to DCs, such as DC-SIGN. DC-SIGN is also expressed on macrophages and Kupffer cells, and although a true murine homolog has not been identified, mouse models expressing human DC-SIGN under the CD11c promoter or immunodeficient mice reconstituted with human immune cells have been used to study its potential as an antigen delivery target [20,115,116,117]. These models have demonstrated that antigen delivered via DC-SIGN can promote efficient antigen uptake, cross-presentation, and enhanced T cell responses [115,116,117]. Similarly, the mannose receptor (CD206), also expressed on macrophages and endothelial cells among others, has been exploited for antigen delivery to DCs [20,118]. Collectively, these findings highlight the flexibility of DC-targeted antigen delivery strategies. Depending on the DC subset or receptor targeted, antigen uptake, intracellular trafficking, presentation, and downstream immune responses can be differentially affected, allowing the approach to be precisely tailored toward specific immunological outcomes (Figure 2).

Beyond the direct use of DC-targeted mAbs, recombinant mAbs bearing antigen have also been encoded and delivered as DNA vaccines. This idea was explored by Nchinda and colleagues with a DNA vaccine encoding a DEC-205-specific scFv fused to HIV gag p41, which significantly improved protection upon challenge with vaccinia virus expressing gag p41 over a nontargeted DNA vaccine [119]. Other studies have expanded this concept to explore a variety of immunological settings. An experimental DNA vaccine that encoded a DEC-205-specific scFv linked to the extracellular domain of HER2 was shown to slow the development of mammary carcinomas in mice [120]. Similar strategies have also been applied to induce protection against botulinum toxin, dengue virus, and hepatitis B virus [121,122,123]. Of note, it has been shown that, in certain contexts, like with direct mAb vaccines, DC-targeted DNA vaccines may induce tolerance rather than protection, highlighting again the crucial importance of adjuvant choice in vaccine design [124].

Vaccines targeting particulate antigens, such as whole viruses or bacteria, can be desirable because they preserve a range of pathogen-associated antigens in their structural context, and DC-targeted mAb-based strategies have been explored for this approach [125]. A recent report described a bispecific nanobody wherein one arm bound DCs and the other targeted foot-and-mouth disease virus (FMDV) [126]. Following incubation of the bispecific nanobody with FMDV, mice vaccinated with this mixture induced cellular and humoral immunity to FMDV [126]. Another bispecific approach targeting the receptor-binding domain (RBD) of SARS-CoV-2 and DNGR-1 prevented death in mice infected with SARS-CoV-2 by targeting neutralized virions to cDC1s, promoting cross-presentation [127]. These results highlight that using DC-targeted mAb-derived molecules to deliver particulate antigens represents an avenue for further exploration.

While DC-targeted recombinant mAbs have many favorable features for vaccine development, it is also important to consider their limitations. For an Ab vaccine to be developed and produced, it must have sufficient stability and solubility [128]. Exposed hydrophobic regions, conformational instability, and heterogeneity of surface charge distribution in either the Ab or the antigenic cargo can lead to self-association and aggregation of the molecule [128]. These properties can also affect the scalability of Ab vaccine manufacturing, which is an important practical consideration for vaccine development [128,129,130]. Aggregation-prone molecules may be more difficult to express and purify with a high yield and consistent quality, and different immunoglobulins may require specific purification strategies [128,131,132]. Therefore, strategies to determine and optimize the biophysical properties of mAbs are critical to successful vaccine development [128,130]. Recent work described the episomal stable production of mAb-antigen fusion immunoglobulins in Expi293 cell pools, yielding approximately 25–30 mg/L, compared to transient transfections of similar constructs that yield 5–7 mg/L [133]. This may provide a promising starting point for further optimization toward scalable manufacturing of mAb vaccines. Furthermore, while the range of choices of specificity, Ab backbone, linkage mechanism, and antigen can be beneficial, they also require careful consideration and empirical optimization for specific immunological settings. For example, different antigenic cargos may vary in their sensitivity to lysosomal degradation, impacting antigen processing and cross-presentation [134]. Additionally, translation of mAb vaccines from animal models to humans must consider the need for species-appropriate targeting Abs and differences in the distribution and expression levels of target receptors [51,135].

## 3. Applications of Recombinant Antibody Vaccines

### 3.1. Antiviral Vaccines

Given the tunable nature of recombinant mAb systems, these approaches have been explored in a variety of vaccination settings. Among the most extensively studied applications of recombinant mAb vaccines is the induction of protective immunity against viral pathogens. Vaccines against human immunodeficiency virus (HIV) are a major focus of vaccine development, and an experimental vaccine delivering HIV antigens to DCs was initially developed in 2006 using an anti-DEC-205 mAb engineered with HIV gag p24 [72]. The vaccine, administered in combination with an agonistic anti-CD40 mAb and poly (I:C), induced CD4^+^ T cell responses, including cytokine production, mucosal responses, and T cell memory in a mouse model, and was later shown to also enable cross-presentation and stimulation of CD8^+^ T cells in vitro [72,136]. This approach was shown to elicit mucosal and systemic responses in mice when administered intranasally with the adjuvant poly-ICLC [73]. More recently, strategies have been explored targeting various HIV antigens via anti-CD40 mAbs, often in conjunction with poly-ICLC [74,75,77,112]. These strategies have demonstrated potential to elicit both cellular and humoral immune responses in rhesus macaques and HIV-uninfected humans [74,75,77]. Furthermore, an anti-Langerin mAb delivering HIV Env was shown to elicit a humoral response in mice including production of neutralizing antibodies without the aid of an adjuvant, which could eventually offer additional clinical utility [101,137].

SARS-CoV-2 has become another important target for recombinant mAb vaccine development. Anti-CD40 mAbs have also been used for SARS-CoV-2 vaccines [76,114]. One such platform delivered the RBD of the SARS-CoV-2 spike protein to DCs, while another delivered several highly conserved epitopes from the spike and nucleocapsid proteins, with both approaches enhancing protective immune responses in animal models [76,114]. A vaccine delivering RBD to Clec9a likewise elicited humoral and cellular immune responses in mice, including mucosal immune responses [13].

Various strategies have also been applied for vaccination against Epstein–Barr virus (EBV). Initially, an experimental anti-DEC-205 mAb carrying EBV nuclear antigen 1 (EBNA1) was shown to expand effector and memory T cells and elicit anti-EBNA1 antibodies in some cases in vitro [138]. The anti-DEC-205-EBNA1 recombinant mAb has been further investigated as part of a heterologous prime-boost vaccination series in combination with other vaccine types [139]. Additional approaches have employed alternative targeting receptors, such as Langerin, or alternative antigens, including the immediate-early protein BZLF1 [100,140].

Influenza has been another target investigated for mAb vaccine approaches. Due to the antigenic diversity among influenza A virus subtypes, efforts to develop “universal” influenza vaccines have focused on highly conserved antigens such as the ectodomain of the matrix-2 protein (M2e) [141,142]. A DEC-205 mAb M2e vaccine resulted in robust heterosubtypic protection against mortality in mice challenged intranasally with live virus [143]. However, another study found that in chickens, anti-CD11c scFv delivering hemagglutinin to DCs elicited more robust immune responses in comparison to anti-DEC205 scFv [144]. Intranasal recombinant mAb-mediated antigen delivery has been evaluated in a model using ovalbumin (OVA) antigen and challenge with Flu-OVA influenza virus [145]. Targeting to either DEC-205 or Clec12a induced protective immune responses in this system, including the generation of tissue-resident memory CD8^+^ T cells [145]. Recombinant mAb vaccines have additionally been experimentally evaluated for use against ebolavirus (EBOV). A recent study showed that immunization with anti-DEC-205 mAb fused to EBOV nucleoprotein (NP) decreased viremia and increased anti-NP antibody production following challenge with EBOV relative to unvaccinated mice [146]. These studies collectively show that recombinant mAb vaccines may warrant further investigation for prevention of viral disease.

Because infection with human papillomavirus (HPV) can cause malignant transformation, vaccinations against HPV sit at the critical intersection of antiviral and anticancer vaccine development [147,148]. As the HPV oncoproteins E6 and E7 are selectively expressed by HPV-infected malignant cells, they represent ideal targets for antitumor vaccine development with limited potential for inducing autoimmunity [148,149]. Selective delivery of E7 via DEC205 mAb or scFv alongside Poly (I:C) or PD-L1 blockade, respectively, induced antitumor responses in mice [46,78]. A similar approach targeted to CD40 led to increased frequency of HPV-specific CD8^+^ T cells infiltrating into tumors in an experimental model [113].

### 3.2. Vaccines Against Other Infectious Agents

Recombinant mAb vaccines have also been developed against other infectious agents in addition to viruses. An early example is the development of DC-targeted delivery of *Yersinia pestis* antigens. An early experimental vaccine strategy involved the *Y. pestis* LcrV protein targeted via anti-DEC-205 monoclonal antibody and was able to induce antigen-specific CD4^+^ T cell responses in mice [150]. This approach was also able to provide protective mucosal immunity in a murine model when delivered via an intranasal route [151]. Another study indicated that DCIR2 targeting was even more effective than DEC-205 targeting in promoting survival of mice following challenge with *Y. pestis* [104].

DC-targeted recombinant mAb vaccines may also be useful for targeting intracellular bacteria. One experimental strategy targeted OVA to DC-SIGN in a transgenic mouse model expressing DC-SIGN under the CD11c promoter [117]. Vaccination with anti-DC-SIGN conjugated to OVA enhanced with anti-CD40 antibody led to significantly decreased bacterial burden after challenge with OVA-expressing *Listeria monocytogenes*, both when challenged 7 or 35 days after vaccination [117]. As *L. monocytogenes* can be especially dangerous for neonates, an experimental strategy targeting the Clec9a^+^ DC population that is abundant in the first few days of life was developed [79]. Vaccination of neonates with anti-Clec9a-OVA along with poly (I:C) was found to enhance protection against OVA-expressing *L. monocytogenes* later in life [79]. While these studies provide a necessary foundation for developing recombinant mAb vaccines against intracellular bacteria, future work is still needed to determine their efficacy when applied to biologically relevant *L. monocytogenes* antigens.

Another intracellular bacterium that has been a target of interest is *Mycobacterium tuberculosis*. Attempts to develop mAb vaccine strategies for *M. tuberculosis* have had mixed results in experimental models. Some studies have shown that delivering antigen via DEC-205- or DC-SIGN-targeted mAb failed to improve protection or bacterial control upon challenge [152,153]. However, other studies that vaccinated via the intranasal route, rather than subcutaneously or intraperitoneally, have shown significant improvements in controlling bacterial load [154,155]. More recently, an approach aiming to improve the existing *M. tuberculosis* vaccine, Bacillus Calmette-Guérin (BCG), by developing a recombinant strain expressing a DEC-205-specific scFv was developed [156]. This strategy yielded significantly more protection in mice than vaccination with BCG alone, at both four and twenty weeks post-challenge [156].

In addition to vaccines against bacterial infections, DC-targeted mAb vaccines have also been investigated against protozoa including *Leishmania major* and *Toxoplasma gondii*. Delivery of the stress-inducible 1 protein of *L. major* via an anti-DEC-205 mAb induced more interferon-γ-producing CD4^+^ T cells and significantly reduced the number of parasites after challenge in a mouse model [157]. Similarly, an experimental vaccine targeting the *T. gondii* surface antigen SAG1 to DCs via an anti-DEC-205 scFv demonstrated significant improvement of cellular and humoral immune responses, along with a reduction in brain parasite burden, using both intranasal and subcutaneous administration [158]. These studies collectively demonstrate that mAb vaccines are useful against a broad range of pathogens relevant to human health.

### 3.3. Vaccines Against Cancer

Cancer is a significant public health issue and therefore is another important target of vaccine development. Just as in an immune response against infectious agents, DCs are also critical for the priming and activation of T cells for antitumor responses, and therefore, mAb vaccines targeted to DCs have also been investigated in cancer settings [159,160]. Initial studies demonstrated the promise of mAb cancer vaccines using anti-DEC-205 mAb or scFv conjugated to melanoma antigens such as tyrosine-related protein (trp) 2 or glycoprotein (gp) 100, resulting in substantial reductions in tumor growth relative to control mice [39,161]. Clec9a has also been explored as a target receptor for cancer vaccination, and OVA delivery via Clec9a induced significant reductions in lung pseudometastases of OVA-expressing melanoma [92]. Studies have also investigated cancer vaccination strategies focused on the cDC2 subset with DCIR2 mAb and found that DCIR2 targeting provided comparable protection to DEC-205 targeting upon challenge [103]. Furthermore, beyond melanoma models, an experimental cancer vaccine for breast cancer targeting HER2 via DEC-205 mAb led to significantly improved survival of mice after tumor challenge relative to untargeted HER2 [162].

An approach targeting the cancer-testis antigen NY-ESO-1 via DEC-205 demonstrated the capacity to activate human CD4^+^ and CD8^+^ T cells in vitro [118]. Given this promising data, the approach was developed into CDX-1401 vaccine, consisting of a human anti-DEC-205 mAb conjugated to full-length NY-ESO-1 [11]. In phase 1 clinical trials wherein CDX-1401 was co-administered with the adjuvants resiquimod and poly-ICLC, the development of humoral and cellular immune responses w observed in most patients [11]. Clinically, 13 out of 45 patients enrolled in the trial had a stable disease course during treatment, but tumor regression was not observed in most patients [11]. Another trial found that fms-like tyrosine kinase 3 (Flt3) ligand improved humoral and T cell responses to CDX-1401 but did not assess clinical benefit [163]. CDX-1401 was recently evaluated in combination with decitabine and nivolumab therapy, and of seven patients who completed a study cycle, six exhibited either a complete response or stable disease course, suggesting a modest clinical response [164]. Together, these trials demonstrate the clinical feasibility and safety of mAb-mediated cancer vaccines and highlight the need for continued investigation into their potential role as components of immunotherapy regimens.

There are other ongoing efforts to find alternative strategies to augment the efficacy of mAb cancer vaccines. Recently, an experimental multi-epitope vaccine was developed, delivering gp100 and trp2 to DCs simultaneously via an anti-DEC-205 mAb containing both antigens [48]. Another study described a novel modular vaccine platform in which antigens can be linked to an anti-DEC-205 scFv via proximity-induced conjugation, enabling the vaccine to be readily adapted for delivery of various tumor neoepitopes [12]. Anti-DEC-205 mAb vaccines may also be leveraged as part of prime-boost vaccination in conjunction with oncolytic rhabdovirus, and this approach has been shown to improve survival in mice with B16-OVA tumors [165]. Ultimately, these studies suggest that DC-targeted mAb vaccines are a promising area for future research as cancer therapeutics, with many possible strategies to improve their efficacy currently being studied. Experimental vaccines targeting biologically relevant antigens are summarized in Table 1.

### 3.4. Inverse Vaccination to Prevent Autoimmune Disease

Because the outcome of antigen presentation depends on the larger immunological context, the same principles of antigen delivery can be used in the steady state to tolerize the immune system to a particular antigen [4,18,19]. Strategies to ameliorate autoimmune disease with monoclonal mAb have been extensively reviewed by Iberg et al. and have been experimentally evaluated for prevention of diseases including animal models of multiple sclerosis, type 1 diabetes, arthritis, inflammatory bowel disease, and uveoretinitis [18]. Collectively, this research reinforces the context-dependent nature of DC-targeted antigen delivery and highlights the potential to restore tolerance with vaccines, rather than promoting protective immunity.

## 4. Using Monoclonal Antibodies to Augment Other Vaccination Strategies

Strategies derived from recombinant mAb vaccines can also be used to enhance other vaccination strategies by pinpointing delivery of the vaccine to relevant cells. One such use that has been explored is the improvement of mRNA-LNP vaccines. A recent publication described the use of bispecific antibodies, with one arm of the antibody targeting polyethylene glycol on the surface of the LNP, and the other arm specific to a receptor on the target cell population, in this case, epidermal growth factor receptor or folate hydrolase 1 [166]. This approach led to a significant uptake of the vaccine within tumor xenografts expressing the targeted receptors, and a significant decrease in uptake by the liver, a common site for off-target accumulation of mRNA-LNP vaccines [166]. Another recent study combined this idea with the principles of DC targeting using an neoantigen-delivering LNP conjugated with a Clec9a-specific nanobody, leading to enhanced T cell responses and decreased tumor volume in a mouse model of Lewis lung carcinoma [47]. Furthermore, anti-DEC-205 antibodies were also shown to assist in delivering mRNA-LNP cancer vaccines to DCs [167]. This strategy integrates the benefits of DC-targeted mAb vaccination with already modular mRNA-LNP-based vaccines, potentially enabling highly personalized and effective cancer vaccines to be developed.

Adenoviral vector vaccines have also been shown to benefit from mAb-assisted DC targeting. Early research leveraged bispecific antibodies targeting both the adenoviral capsid and CD40 to enhance transduction of DCs [168,169]. More recently, approaches involving incorporating DC-specific antibodies directly into the adenovirus itself, altering the tropism of the vector, have been explored [170,171]. Finally, DC-targeting mAb can be used to enable targeted adjuvant delivery. A recent publication describes anti-Clec9a mAb used to precisely deliver CpG oligonucleotides to cDC1, leading to activation of Toll-like receptor 9 and enhancement of in vivo vaccine responses [172]. Overall, these studies highlight a range of applications for mAb not just as vaccines themselves, but also to augment the efficacy and specificity of other vaccination strategies.

## 5. Conclusions

Recombinant mAb-based antigen delivery platforms have the potential to act as vaccines across a broad spectrum of immunological settings. Because both the mAb specificity and antigen cargo can be independently modified, these approaches provide a modular framework for vaccine design that builds on well-established principles of antigen uptake, processing, and presentation by DCs. Targeting of specific DC subsets allows for shaping of downstream immunological outcomes, and co-administration with specific adjuvants can further tune the magnitude and quality of the response. The potential of these vaccines has been evaluated in preclinical and clinical settings for use against viruses, bacteria, protozoa, cancer, and to prevent autoimmune disease. Furthermore, recombinant mAb targeting can be incorporated into other vaccine platforms, highlighting the versatility of this strategy. Despite substantial progress, considerable untapped potential remains in the field of recombinant mAb vaccine development. While the current body of research demonstrates that recombinant mAb vaccines are an adaptable platform for initiating and directing immune responses, future work should focus on defining the combinations of receptor targets, antigens, and adjuvants most likely to support clinical translation. Additionally, approaches that have demonstrated success in experimental models may require further optimization to support human translation and scalable manufacturing. Continued refinement of these approaches may expand their potential for future vaccine development.

## Figures and Tables

**Table 1:** Summary of experimental vaccines using biologically relevant antigens.

Receptor	Disease	Antigen	Organism	Immune Response Induced	References
CD11c	Influenza	Hemagglutinin	Chickens	Humoral	[144]
CD40	HIV	Env gp140	Rhesus macaques; humans	Cellular and humoral	[74,75]
		Multiple	Rhesus macaques	Cellular and humoral	[77,112]
	HPV	E6 and E7	Mice	Cellular	[113]
	SARS-CoV-2	RBD	Rhesus macaques	Cellular and humoral	[76]
		Multiple	Mice	Humoral	[114]
Clec9a	SARS-CoV-2	RBD	Mice	Cellular and humoral	[13]
DCIR2	*Y. pestis*	LcrV	Mice	Cellular and humoral	[104]
DEC-205	Breast cancer	HER2/neu	Mice	Cellular and humoral	[162]
	Ebolavirus	Nucleoprotein	Mice	Cellular and humoral	[146]
	EBV	BZLF1	Mice	Cellular	[140]
		EBNA1	Mice	Cellular and humoral	[138,139]
	HIV	gag p24	Mice	Cellular	[72]
	HPV	E7	Mice	Cellular	[46,78]
	Influenza	Hemagglutinin	Chickens	Humoral	[144]
		M2e	Mice	Humoral	[143]
	*L. major*	LmST1a	Mice	Cellular	[157]
	Melanoma	gp100	Mice	Cellular	[39]
		TRP-2	Mice	Cellular	[161]
	*M. tuberculosis*	ESAT-6	Mice	Cellular	[154,155]
	*T. gondii*	SAG1	Mice	Cellular and humoral	[158]
	Various cancers	NY-ESO-1	Humans	Cellular and humoral	[11,118,163,164]
	*Y. pestis*	LcrV	Mice	Cellular and humoral	[104,150,151]
Langerin	HIV	Env gp140	Mice; rabbits	Cellular and humoral	[100,101,137]

## Data Availability

No new data were created or analyzed in this study. Data sharing is not applicable to this article.

## Abbreviations

The following abbreviations are used in this manuscript:

Ab	Antibody
APC	Antigen-presenting cell
BCG	Bacillus Calmette-Guérin
BDCA2	Blood dendritic cell antigen 2
cDC	Conventional dendritic cell
DC	Dendritic cell
DCIR2	Dendritic cell inhibitory receptor 2
EBNA1	EBV nuclear antigen 1
EBOV	Ebolavirus
EBV	Epstein–Barr virus
FcRn	Neonatal Fc receptor for IgG
Flt3	Fms-like tyrosine kinase 3
Gp	Glycoprotein
HIV	Human immunodeficiency virus
HPV	Human papillomavirus
LC	Langerhans cell
*L. major*	*Leishmania major*
*L. monocytogenes*	*Listeria monocytogenes*
LNP	Lipid nanoparticle
M2e	Matrix-2 protein
mAb	Monoclonal antibody
MHC	Major histocompatibility complex
NP	Nucleoprotein
OMV	Outer membrane vesicle
OVA	Ovalbumin
pDC	Plasmacytoid dendritic cell
Poly(I:C)	Polyinosinic:polycytidylic acid
PRR	Pattern recognition receptor
RBD	Receptor-binding domain
SARS-CoV-2	Severe acute respiratory syndrome coronavirus 2
scFv	Single-chain variable fragment
Siglec-H	Sialic acid binding Ig-like lectin H
*T. gondii*	*Toxoplasma gondii*
TLR	Toll-like receptor
T_reg_	Regulatory T cell
Treml4	Trem-like 4
Trp	Tyrosine-related protein
T_STEM_	Stem-like T cell
*Y. pestis*	*Yersinia pestis*
